# Supplementary material for: Transcriptomic changes including p53 dysregulation prime DNMT3A mutant cells for transformation
Source: EMBO Rep. 2025 Apr 30;26(11):2855–82. doi: 10.1038/s44319-025-00450-4 (PMC12152194; doi:10.1038/s44319-025-00450-4)
Supplement: Supplementary file 1 — Table EV1 [file 44319_2025_450_MOESM1_ESM.docx]

**Table EV1: Defining cell markers for cell subsets**

| **Cell Type** | **Cell Markers** |
| --- | --- |
| LT-HSC | Lin-SCA1+cKIT+CD135/Flk2-CD34-;  Lin-SCA1+cKIT+CD135/Flk2-CD150+CD48- |
| ST-HSC | Lin-SCA1+cKIT+CD135/Flk2+CD34+; Lin-SCA1+cKIT+CD135/Flk2-CD150-CD48- |
| MPP | Lin-SCA1+cKIT+CD135/Flk2+CD34+ |
| MPP2 | Lin-SCA1+cKIT+CD135/Flk2-CD150+CD48+ |
| MPP3 | Lin-SCA1+cKIT+CD135/Flk2-CD150-CD48+ |
| LSK | Lin-SCA1+cKIT+ |
| CLP | Lin-SCA1midcKITmid |
| GMP | Lin-SCA1-cKIT+CD16/32+CD34+ |
| CMP | Lin-SCA1-cKIT+CD16/32-CD34+ |
| MEP | Lin-SCA1-cKIT+CD16/32-CD34- |
| Granulocytes | TCRβ-B220-GR1+MAC1+ |
| Macrophages | TCRβ-B220-GR1-MAC1+ |
| Pro-pre B | TCRβ-B220midIgM- |
| Immature B | TCRβ-B220midIgMmid |
| Transitional B | TCRβ-B220+IgM+ |
| Mature B | TCRβ-B220+IgD+ |
| DN | CD4-CD8- |
| DN1 | CD4-CD8-CD25-CD44+ |
| DN2 | CD4-CD8-CD25+CD44+ |
| DN3 | CD4-CD8-CD25+CD44- |
| DN4 | CD4-CD8-CD25-CD44- |
| DP | CD4+CD8+ |
| CD4+ | CD4+CD8- |
| CD8+ | CD4-CD8+ |
| Lineage | CD4, CD8, CD11b/MAC1, TER119, Ly6, B220, CD2, CD3, CD19, F4/80, NK1.1, GR1 |
