# Supplementary material for: Transcriptomic changes including p53 dysregulation prime DNMT3A mutant cells for transformation
Source: EMBO Rep. 2025 Apr 30;26(11):2855–82. doi: 10.1038/s44319-025-00450-4 (PMC12152194; doi:10.1038/s44319-025-00450-4)
Supplement: Supplementary file 3 — Expanded View Figures [file 44319_2025_450_MOESM3_ESM.pdf]

Expanded View Figures

Figure EV1. Phenotyping of the *Dnmt3a*$^{R878H/+}$ mutant mice.

(**A**) Representative flow cytometry plots of spleen cells from *Dnmt3a*$^{R878H/+}$ mutant mice and WT littermates. Leukocytes were gated by FSC-A and SSC-A, then cell doublets were gated out by FSC-H and FSC-A (not shown). Live cells were gates as being negative for the dead cell marker used, i.e. PI, DAPI, or Fluorogold. Cell subsets were identified based on their expression of cell type specific surface markers: CD4 + T cells = TCRβ + /CD4 + /CD8-; CD8 + T cells = TCRβ + /CD4-/CD8+); transitional B cells = B220 + /IgD$_{low}$/IgM$_{high}$; mature B cells = B220 + /IgD + /IgM$_{mid}$; granulocytes = TCRβ-/B220-/GR1 +/MAC1+; and macrophages = TCRβ-/B220-/GR1-/MAC1+. (**B**) Percentages of the indicated haematopoietic cell subsets determined by flow cytometry in the blood and spleen of mice of the indicated genotype. (**C–H**) Blood cell composition in mice with γ-irradiation induced thymic T cell lymphoma: lymphocytes (**C**), neutrophils (**D**), monocytes (**E**), eosinophils (**F**), basophils (**G**), and large unstained cells (LUCs) (**H**) determined by ADVIA analysis. (**I**) Flow cytometry plots of TCRβ and B220 expression on spleen cells from thymic T lymphoma burdened *Dnmt3a*$^{R878H/+}$ mutant mice and WT littermates collected at ethical endpoint. Leukocytes were gated by FSC-A and SSC-A, then cell doublets were gated out by FSC-H and FSC-A (not shown). Live cells were gated as being negative for the dead cell marker used, i.e. PI, DAPI, or Fluorogold. (**J**) Flow cytometry plots of TCRβ and B220 expression in thymus samples from thymic T lymphoma burdened *Dnmt3a*$^{R878H/+}$ mice and WT littermates collected at the ethical endpoint. Leukocytes were gated by FSC-A and SSC-A, then cell doublets were gated out by FSC-H and FSC-A (not shown). Live cells were gates as being negative for dead cell markers PI, DAPI, or Fluorogold. (**K**) Graphical representations of the proportions of TCRβ and B220 expression in thymus of thymic T cell lymphoma burdened *Dnmt3a*$^{R878H/+}$ mice and WT littermates represented in (**J**). Data information: Error bars are the mean (±SEM) of (**B**) $n = 4$ *Dnmt3a*$^{R878H/+}$ and $n = 5$ *Dnmt3a*$^{+/+}$ (blood), $n = 6$ *Dnmt3a*$^{R878H/+}$ and $n = 6$ *Dnmt3a*$^{+/+}$ (spleen), (**C–H**) $n = 7$ *Dnmt3a*$^{R878H/+}$ and $n = 8$ *Dnmt3a*$^{+/+}$, and (**K**) $n = 7$ *Dnmt3a*$^{R878H/+}$ and $n = 5$ *Dnmt3a*$^{+/+}$ independent biological repeats. Statistical significance was assessed using Prism 10 software by t tests. ns, not significant; $p > 0.05$, *$p < 0.05$.

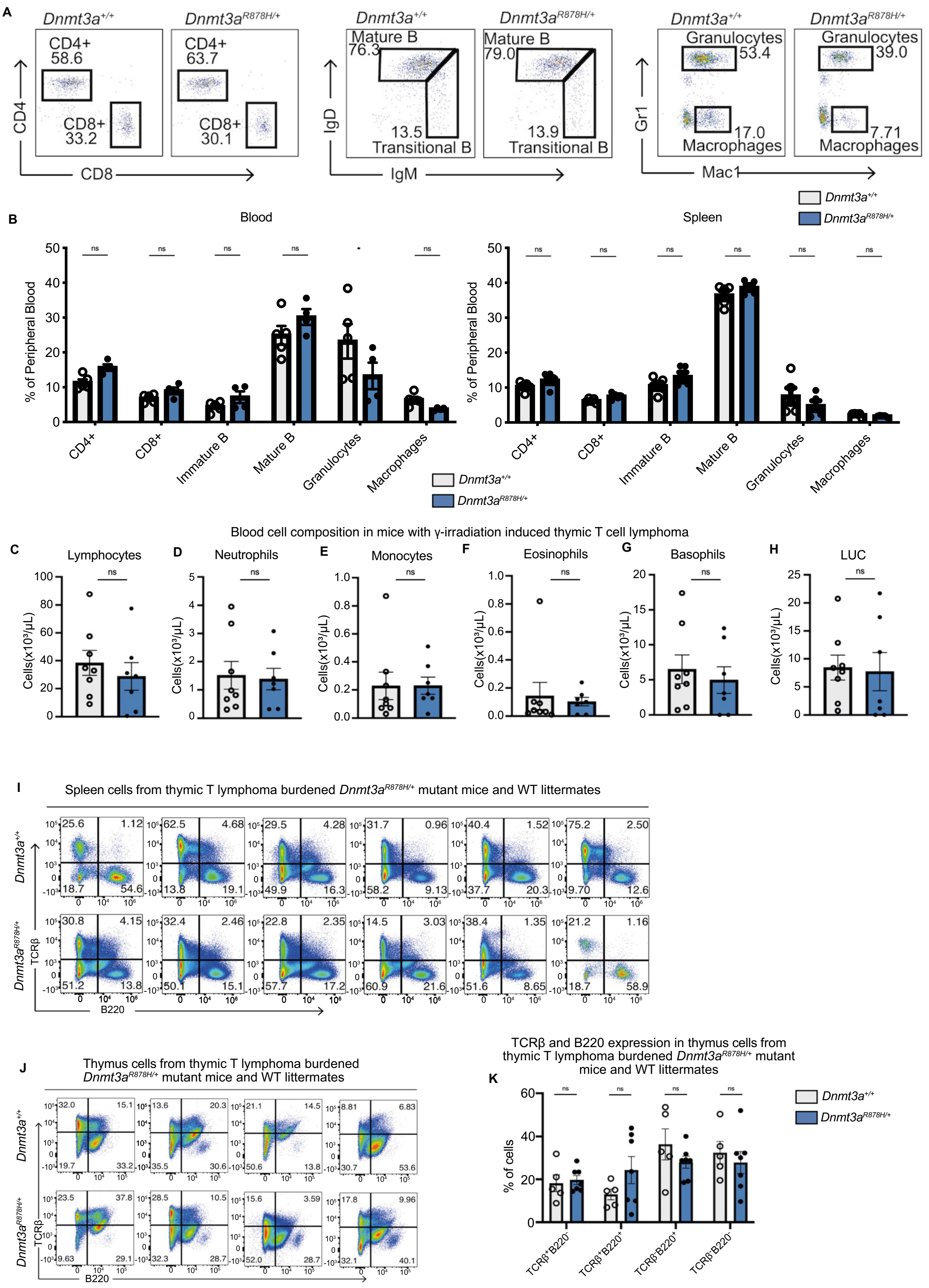
A
Dnmt3a+/+
Dnmt3aR878H/+
CD4+ 58.6
CD8+ 33.2
CD4+ 63.7
CD8+ 30.1
CD4
CD8
Mature B 76.3
13.5 Transitional B
Mature B 79.0
13.9 Transitional B
IgD
IgM
Granulocytes 53.4
17.0 Macrophages
Granulocytes 39.0
7.71 Macrophages
Gr1
Mac1
B
Blood
Spleen
% of Peripheral Blood
CD4+
CD8+
Immature B
Mature B
Granulocytes
Macrophages
ns
Blood cell composition in mice with γ-irradiation induced thymic T cell lymphoma
C Lymphocytes
D Neutrophils
E Monocytes
F Eosinophils
G Basophils
H LUC
Cells(x10³/μL)
I
Spleen cells from thymic T lymphoma burdened Dnmt3aR878H/+ mutant mice and WT littermates
TCRβ
B220
J
Thymus cells from thymic T lymphoma burdened Dnmt3aR878H/+ mutant mice and WT littermates
K
TCRβ and B220 expression in thymus cells from thymic T lymphoma burdened Dnmt3aR878H/+ mutant mice and WT littermates
% of cells
TCRβ+B220-
TCRβ+B220+
TCRβ-B220+
TCRβ-B220-

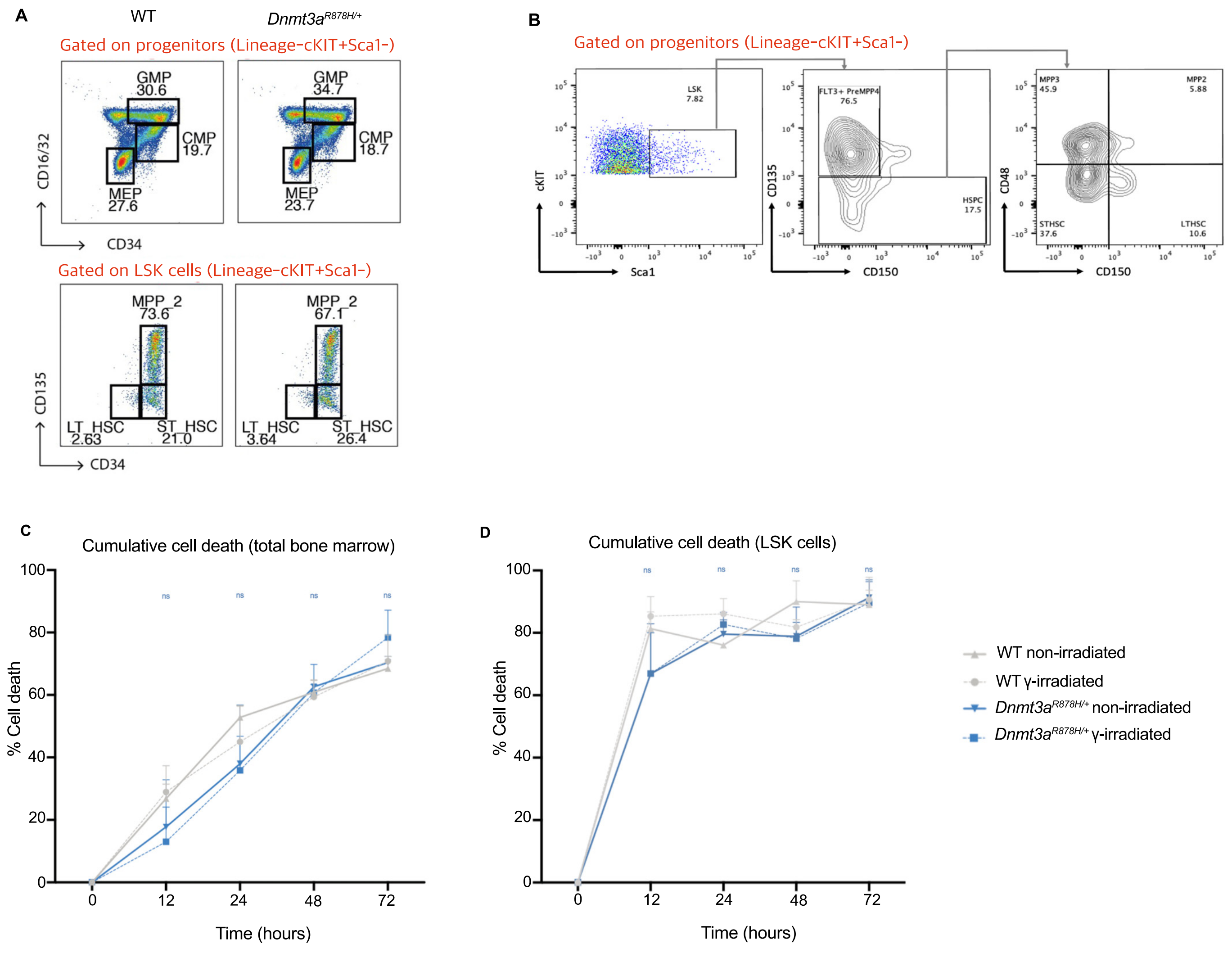

Figure EV2. Flow cytometry gating strategies and ex vivo γ-irradiation induced cell death analysis.

(**A**) Flow cytometry gating strategy to identify HSPCs amongst total bone marrow cells in mice. Granulocyte myeloid progenitors (GMP), common myeloid progenitors (CMP), megakaryocyte erythroid progenitors (MEP), multipotent progenitor cells (MPP), long-term haematopoietic stem cells (LT-HSC), short-term haematopoietic stem cells (ST-HSC). (**B**) Flow cytometric gating strategy to define the different cell subsets in the HSPC compartment when assessing γ-irradiation-induced cell death. Long-term haematopoietic stem cells (LT-HSC), short-term haematopoietic stem cells (ST-HSC), megakaryocyte-biased multipotent progenitor subset (MPP2), and myeloid-biased multipotent progenitor subset (MPP3) multipotent progenitor cells 3. (**C**, **D**) Representations of cumulative cell death from flow cytometric analyses of (**C**) total cells and (**D**) LSK cells from the bone marrow of *Dnmt3a*$^{R878H/+}$ mutant mice and WT littermates, following 1.5 Gy γ-irradiation or untreated controls. Percentage of cell death is presented relative to time point 0. Data information: (**C**, **D**) Error bars are the mean (±SEM). The experiment was performed with three independent biological replicates ($n = 3$ *Dnmt3a*$^{R878H/+}$ and $n = 3$ *Dnmt3a*$^{+/+}$ γ-irradiated mice, and $n = 3$ *Dnmt3a*$^{R878H/+}$ and $n = 3$ *Dnmt3a*$^{+/+}$ non-irradiated mice) with three technical replicates, resulting in a total of 9 mice per genotype and treatment. Statistical significance was assessed using Prism 10 software by 2-way ANOVA with Šídák's multiple comparisons test; ns, not significant; $p > 0.05$.

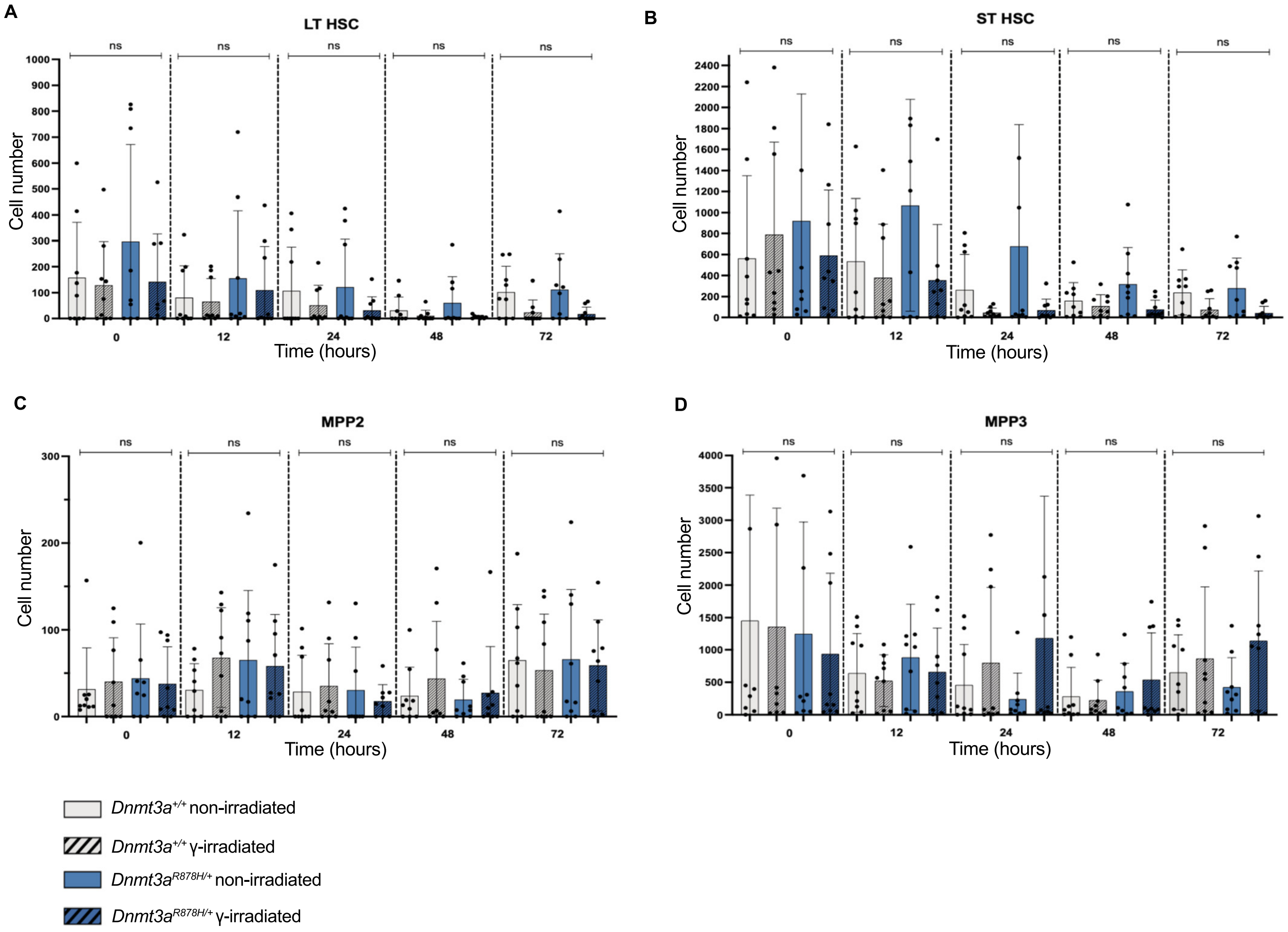

Figure EV3. Ex vivo irradiation induced cell death analysis.

Bone marrow was harvested from 8–12-week-old mice and left untreated, or given 1.5 Gy γ-irradiation immediately after harvesting. These cells were placed in culture and measured by flow cytometry at baseline, 12-, 24-, 48- and 72-h timepoints. (**A–D**) Flow cytometric analyses of the HSPC compartment were used to determine the cell numbers at each time point for long-term haematopoietic stem cells (LT-HSC) (**A**), short-term haematopoietic stem cells (ST-HSC) (**B**), megakaryocyte-biased multipotent progenitor subset (MPP2) (**C**), and myeloid-biased multipotent progenitor subset (MPP3) (**D**). For each condition, $n = 9$ mice (3 mice per treatment and genotype across 3 independent experiments). Data are presented as mean +/− SEM. Data information: Error bars are the mean (±SEM). The experiment was performed with three independent biological replicates ($n = 3$ *Dnmt3a*$^{R878H/+}$ and $n = 3$ *Dnmt3a*$^{+/+}$ γ-irradiated mice, and $n = 3$ *Dnmt3a*$^{R878H/+}$ and $n = 3$ *Dnmt3a*$^{+/+}$ non-irradiated mice) with three technical replicates, resulting in a total of 9 mice per genotype and treatment. Statistical significance was assessed using Prism 10 software by 2-way ANOVA with Tukey's multiple comparisons test; ns, not significant; $p > 0.05$.

 © The Author(s)

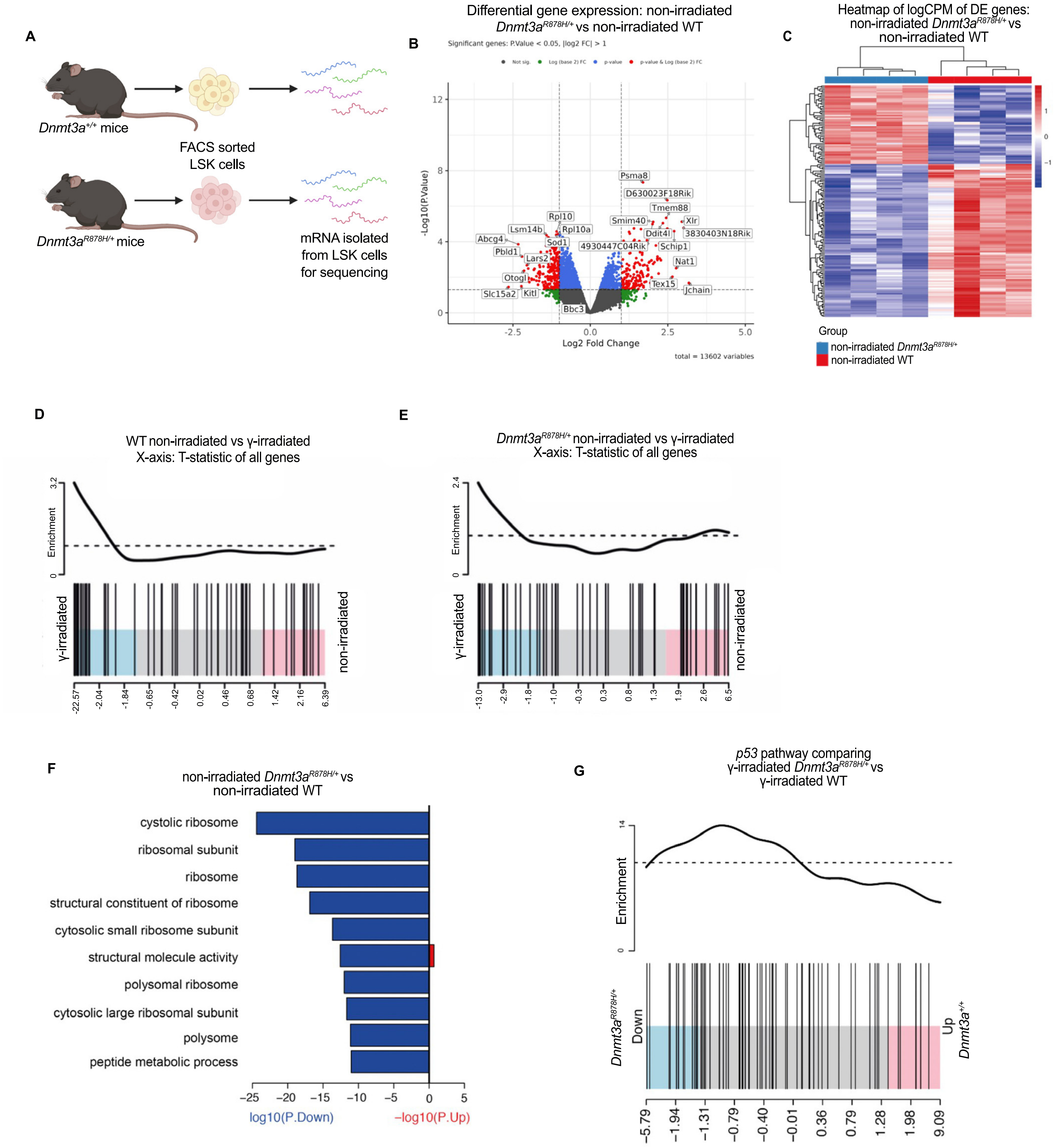
A
Dnmt3a+/+ mice
FACS sorted
LSK cells
Dnmt3aR878H/+ mice
mRNA isolated
from LSK cells
for sequencing
B
Differential gene expression: non-irradiated
Dnmt3aR878H/+ vs non-irradiated WT
Log2 Fold Change
C
Heatmap of logCPM of DE genes:
non-irradiated Dnmt3aR878H/+ vs
non-irradiated WT
Group
non-irradiated Dnmt3aR878H/+
non-irradiated WT
D
WT non-irradiated vs γ-irradiated
X-axis: T-statistic of all genes
Enrichment
γ-irradiated
non-irradiated
E
Dnmt3aR878H/+ non-irradiated vs γ-irradiated
X-axis: T-statistic of all genes
Enrichment
γ-irradiated
non-irradiated
F
non-irradiated Dnmt3aR878H/+ vs
non-irradiated WT
cystolic ribosome
ribosomal subunit
ribosome
structural constituent of ribosome
cytosolic small ribosome subunit
structural molecule activity
polysomal ribosome
cytosolic large ribosomal subunit
polysome
peptide metabolic process
log10(P.Down)
−log10(P.Up)
G
p53 pathway comparing
γ-irradiated Dnmt3aR878H/+ vs
γ-irradiated WT
Enrichment
Dnmt3aR878H/+ Down
Up Dnmt3a+/+

Figure EV4. RNAseq of LSK cells from untreated and γ-irradiated *Dnmt3a*$^{R878H/+}$ mice and WT littermates.

(**A**) Schematic depicting the experimental workflow whereby bone marrow was harvested from untreated age-matched female *Dnmt3a*$^{R878H/+}$ mice and WT littermates. LSK cells were isolated by FACs from the bone marrow, and their mRNA extracted for RNA-seq. (**B**) Volcano plot depicting differences in gene expression between LSK cells harvested from non-irradiated *Dnmt3a*$^{R878H/+}$ mice cells *vs* non-irradiated WT control mice where genes in red are significantly differentially expressed and ranked by *p*-value and log fold change. *Puma/Bbc3* has been highlighted. (**C**) Hierarchical clustering heatmap depicting differentially expressed genes between non-irradiated *Dnmt3a*$^{R878H/+}$ and WT LSK cells. Red and blue squares indicate genes with high or low gene expression levels, respectively. (**D**, **E**) Barcode plots showing enrichment of genes in the p53 signalling pathway (KEGG pathway mmu04115) in non-irradiated WT LSK cells vs γ-irradiated WT LSK cells (**D**) and non-irradiated *Dnmt3a*$^{R878H/+}$ LSK cells vs γ-irradiated *Dnmt3a*$^{R878H/+}$ LSK cells (**E**). (**F**) Gene ontology analysis reveals the top 10 downregulated pathways in untreated *Dnmt3a*$^{R878H/+}$ LSK cells compared to untreated WT LSK cells. (**G**) Barcode plot showing enrichment of genes in the p53 signalling pathway (KEGG pathway mmu04115) in γ-irradiated *Dnmt3a*$^{R878H/+}$ LSK cells compared to γ-irradiated WT LSK cells. Data information: (**B**, **F**) $n = 4$ *Dnmt3a*$^{R878H/+}$ and $n = 4$ *Dnmt3a*$^{+/+}$ independent biological repeats. Statistical significance criteria is $p < 0.05$, $\log_2$FC > 1. Statistical significance was determined using the limma-trend method with empirical Bayes moderation after fitting a linear model to log-transformed counts per million (logCPM). Surrogate variable analysis (SVA) was incorporated to adjust for hidden sources of variation. GO enrichment analysis was conducted using goana.

 © The Author(s)

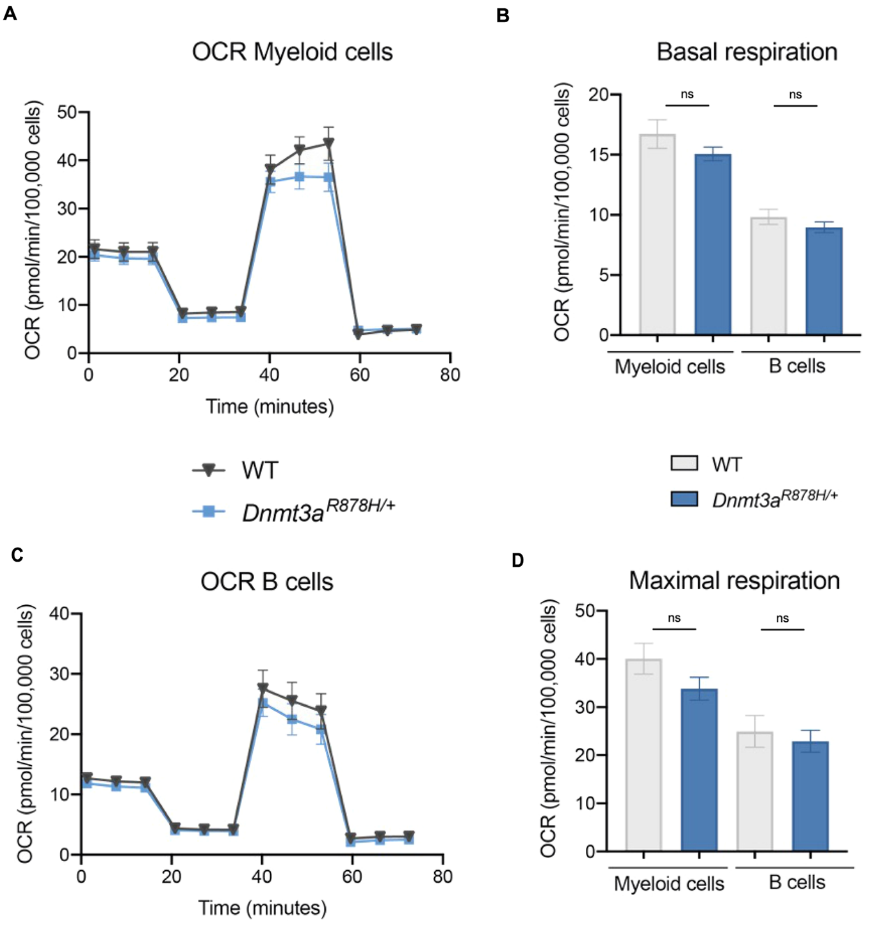

Figure EV5. Oxygen consumption rates of bone marrow cells from *Dnmt3a*$^{R878H/+}$ mutant mice and their WT littermates.

(**A**) Oxygen consumption rate (OCR) profiles of bone marrow-derived myeloid cells from *Dnmt3a*$^{R878H/+}$ or *Dnmt3a*$^{+/+}$ mice. (**B**) OCR profile of B lymphoid cells from the bone marrow of *Dnmt3a*$^{R878H/+}$ or *Dnmt3a*$^{+/+}$ mice. (**C**) Basal respiration rates for myeloid cells and B lymphoid cells from the bone marrow of *Dnmt3a*$^{R878H/+}$ or *Dnmt3a*$^{+/+}$ mice. (**D**) Maximal respiration rates for bone marrow-derived myeloid and B cells from *Dnmt3a*$^{R878H/+}$ or *Dnmt3a*$^{+/+}$ mice. Data information: All error bars are the mean (±SEM) of $n = 7$ *Dnmt3a*$^{R878H/+}$ and $n = 5$ *Dnmt3a*$^{+/+}$ independent biological repeats. Statistical significance was assessed using Prism 10 software by t tests. ns, not significant; $p > 0.05$.

A

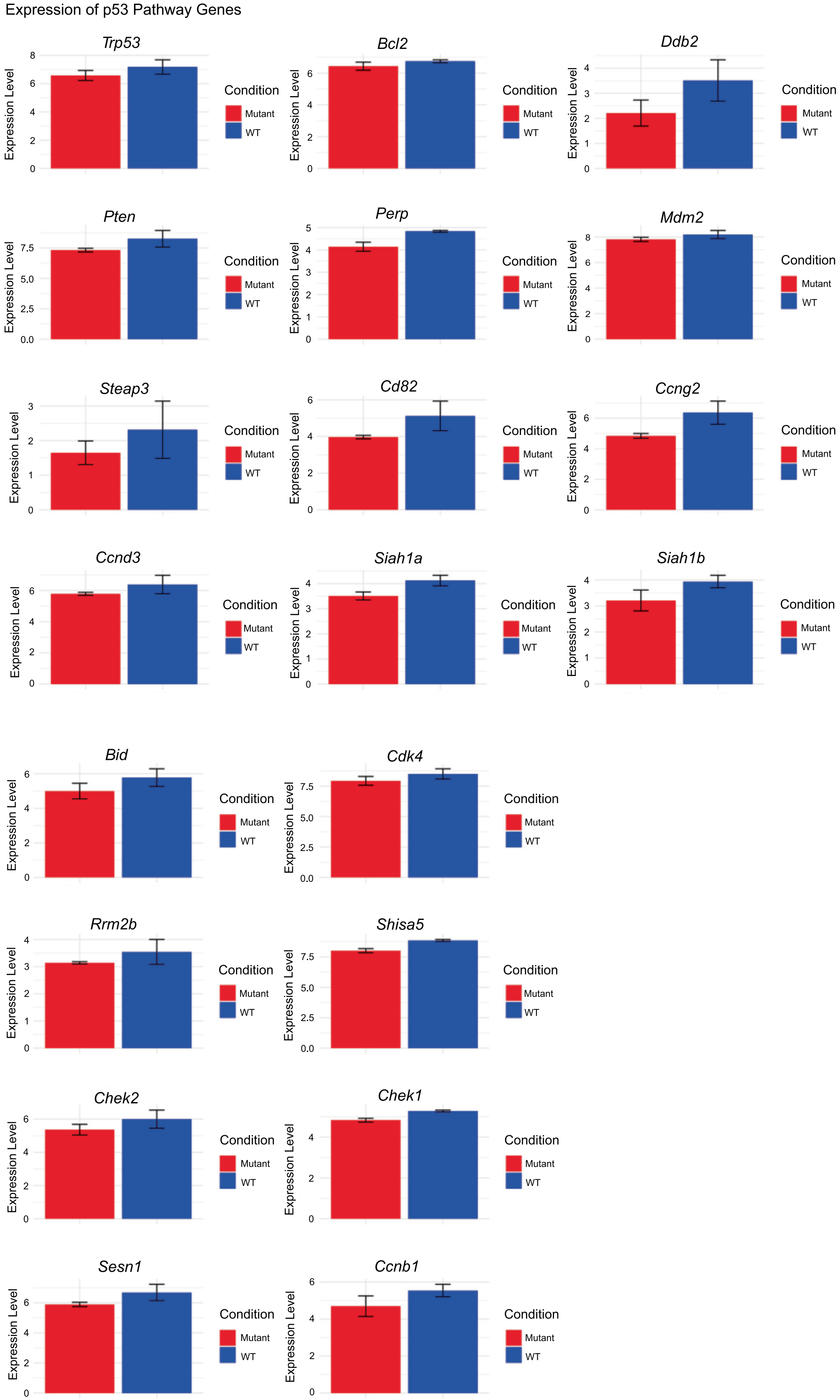

 © The Author(s)

Figure EV6. *p53* pathway genes in LSK cells from *Dnmt3a*$^{R878H/+}$ mice and WT control littermates following 1.5 Gy γ-radiation.

(**A**) RNA-seq analysis of LSK cells from *Dnmt3a*$^{R878H/+}$ mice and WT control littermates following 2 Gy γ-radiation. Bar plots of the relative expression level of p53 pathway genes, where irradiated *Dnmt3a*$^{R878H/+}$ (mutant) cells (red) show lower expression as compared to irradiated WT cells (blue). Data information: All error bars are the mean (±SEM) of $n = 3$ *Dnmt3a*$^{R878H/+}$ and $n = 2$ *Dnmt3a*$^{+/+}$ independent biological repeats. Statistical significance was assessed by t tests. ns, not significant; $p > 0.05$.

© The Author(s)

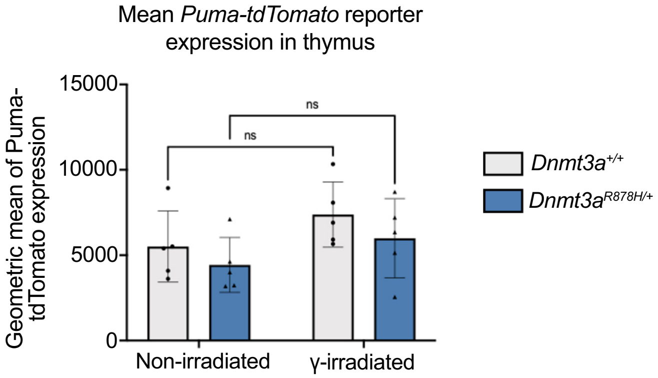

Figure EV7. *Puma-tdTomato* reporter induction in thymocytes following γ-irradiation.

Graphical representation *Puma-tdTomato* expression in the thymocytes of γ-irradiated *Dnmt3a*$^{R878H/+}$/*Puma-tdTomato*$^{KI/+}$ and *Dnmt3a*$^{+/+}$/*Puma-tdTomato*$^{KI/+}$, and non-irradiated *Dnmt3a*$^{R878H/+}$/*Puma-tdTomato*$^{KI/+}$ and *Dnmt3a*$^{+/+}$/*Puma-tdTomato*$^{KI/+}$ mice. Data information: $n = 5$ γ-irradiated *Dnmt3a*$^{R878H/+}$/*Puma-tdTomato*$^{KI/+}$, $n = 5$ non-irradiated *Dnmt3a*$^{R878H/+}$/*Puma-tdTomato*$^{KI/+}$, $n = 5$ γ-irradiated *Dnmt3a*$^{+/+}$/*Puma-tdTomato*$^{KI/+}$, and $n = 5$ non-irradiated *Dnmt3a*$^{+/+}$/*Puma-tdTomato*^{KI} independent biological repeats. Error bars are the mean (±SEM). Statistical significance was assessed using Prism 10 software by 2-way ANOVA with Šídák's multiple comparisons test. ns, not significant; $p > 0.05$.

 © The Author(s)
